# NGAL expression during cardiopulmonary bypass does not predict severity of postoperative acute kidney injury

**DOI:** 10.1186/s12882-017-0479-8

**Published:** 2017-02-21

**Authors:** Martin G. Friedrich, Ioannis Bougioukas, Johanna Kolle, Christian Bireta, Fawad A. Jebran, Marius Placzek, Theodor Tirilomis

**Affiliations:** 1 Department of Thoracic and Cardiovascular Surgery, University Medical Center Göttingen, Robert-Koch-Str. 40, 37075 Göttingen, Germany; 2 Department of Medical Statistics, University Medical Center Göttingen, Göttingen, Germany

**Keywords:** NGAL, Acute renal failure, Kidney injury, Cardiac surgery, Cardiopulmonary bypass

## Abstract

**Background:**

Renal injury is a serious complication after cardiac surgery and therefore, early detection and much more prediction of postoperative kidney injury is desirable. Neutrophil gelatinase-associated lipocalin (NGAL) is a predictive biomarker of acute kidney injury and may increase after cardiopulmonary bypass (CPB). However, time correlation of NGAL expression and severity of renal injury is still unclear. The aim of our study was to investigate CPB-related urine NGAL (uNGAL) secretion in correlation to postoperative renal function.

**Methods:**

Data of NGAL expression along with clinical data of 81 patients (52 male and 29 female) were included in this study. Mean age of the patients was 66.8 ± 12.8 years. Urine NGAL was measured at seven time points (T_0_: baseline; T_1_: start CPB, T_2_: 40 min on CPB; T_3_: 80 min on CPB; T_4_: 120 min on CPB; T_p1_: 15 min after CPB; T_p2_: 4 h after admission to the intensive care unit) and renal function in the postoperative period was classified daily according to Acute Kidney Injury Network (Ronco et al, Int J Artif Organs 30(5): 373–6) criteria (AKIN).

**Results:**

Expression of uNGAL increased at T_4_ (120 min on CPB) and post-CPB (T_p1_ and T_p2_; *p* < 0.01 vs. baseline) but there was no correlation between uNGAL level and duration of CPB nor between uNGAL expression and occurrence of postoperative kidney injury. The renal function over 10 days after surgery remained normal in 50 patients (AKIN level 0), 18 patients (22%) developed mild and insignificant renal injury (AKIN level 1), eight patients (10%) developed moderate renal failure (AKIN level 2), and five patients (6%) severe kidney failure (AKIN level 3). Twenty-four out of 31 patients developed renal failure within the first 48 h after surgery. However, there was no correlation between uNGAL expression and severity of acute renal failure.

**Conclusion:**

Although uNGAL expression increased after CPB, the peak values neither predict acute postoperative kidney injury, nor severity of the injury.

## Background

Renal injury after cardiac surgery is a common complication with significant consequences, regarding prognosis of the patient, intensive care unit stay and therapy costs. The aetiology seems to be multifactorial: low renal blood flow during cardiopulmonary bypass (CPB) may trigger renal failure; several proinflammatory mediators, such as interleukins, TNFα and other metabolites can lead to membrane damage of the renal tubular epithelium [1–6]. The evaluation of renal function is still based on creatinine serum levels, which is simple and less expensive but also with delayed information about acute changes of renal function [7, 8]. The early prediction of the occurrence of acute kidney injury (AKI) after cardiac procedures is of essential importance, as this could restrict further kidney damage and thus improve patient outcome.

Neutrophil gelatinase-associated lipocalin in urine (uNGAL) has been tested as a predictive biomarker of AKI in both experimental and clinical studies [1, 3, 6–8]. In a renal ischemia-reperfusion injury model in the mouse and rat, a marked upregulation of NGAL mRNA and protein levels was seen in the early postischemic period, whereas the appearance of uNGAL in the very first postischemic urine output was related to the duration of ischemia [9]. Woodson et al. found in the rat model increased expression of uNGAL after 30 min of kidney hilum clamping then after 15 and 60 min [10].

Clinical studies of cardiac surgery showed in patients with postoperative AKI increased uNGAL values 2 to 4 h after CPB [1, 6, 7, 11–13]. In most studies, measurements of uNGAL were carried out preoperatively (baseline value), and once again postoperatively at admission of the patients in the intensive care unit (ICU). There are no data of uNGAL during CPB available. The aim of the present study was (1) the measurement of uNGAL expression even during on-pump cardiac surgery, (2) the relationship analysis between uNGAL values and clinical renal outcome, and (3) the analysis of the predictive power of uNGAL on the severity of postoperative AKI.

## Methods

Eighty-one adult patients undergoing cardiac surgery with the use of CPB were included: 34 patients coronary artery bypass grafting (CABG), 19 patients with valve replacements, 22 patients combined CABG and valve replacement, 2 patients with myectomy of hypertrophic cardiomyopathy, 2 patients with aortic reconstruction, and 1 patient with closure of atrial septum defect.

Exclusion criteria were: terminal renal insufficiency, irregular kidney vessels, abnormal kidney morphology (confirmed by ultrasound or magnetic resonance imaging) or preoperative haemodynamic instability.

The preoperative ARF (acute renal failure) score (includes the following 11 preoperative variables: gender, congestive heart failure, left ventricular ejection fraction, use of intra-aortic balloon counterpulsation, chronic lung disease, insulin-requiring diabetes mellitus, previous cardiac surgery, emergency surgery, valve surgery, procedures other than coronary bypass or valve and serum creatinine) was calculated [14].

Urine samples for biomarker analysis were obtained immediately before (T_0_: baseline, before induction of anesthesia), during CPB (T_1_: start CPB; T_2_: 40 min on CPB, T_3_: 80 min on CPB; T_4_: 120 min on CPB) and after CPB (T_P1_: 15 min after CPB; T_P2_: 4 h after admission into ICU (over 5 h after CPB).

The samples were centrifugated and stored in aliquots at −80 °C. The quantitative assessment of uNGAL was performed with the ARCHITECT platform (Chemilumineszenz-Micropartikelimmunoassay (CMIA), Abott GmbH & Co. KG, Wiesbaden, Germany).

Serum creatinine and glomerular filtration rate (eGFR) were routinely measured at baseline (within 72 h before surgery), after admission to the ICU and at least daily in the post-operative period up to 15 days.

The severity of postoperative acute kidney injury was defined and stratified according to the Acute Kidney Injury Network criteria [11, 15]:

- AKIN level 0: no changes
- AKIN level 1: increased serum creatinine for 1.5–2x of the basal value or increased serum creatinine ≥ 0,3 mg/dl or > 25%, reduction of GFR / Urine output < 0.5 mL/kg/h > 6 h
- AKIN level 2: increased serum creatinine for 2–3 of basal value or >50% reduction of GFR / Urine output < 0.5 mL/kg/h > 12 h
- AKIN level 3: increased serum creatinine for > 3 of basal value or >75% reduction of GFR or ein serum creatinine ≥ 4 mg/dl / Urine output < 0.3 mL/kg/h > 24 h or anuria for 12 h

All patients were classified according to these criteria. Since creatinine levels and diuresis over the past 6 h, 12 h , or 24 h is included in calculation of AKIN levels, levels were determined every day postoperatively.

In general, patients with AKIN level 0 or level 1 received no specific therapy, whereas therapy was modified for patients with AKIN level 2 and level 3 (differentiated volume substitution therapy, diuretics or renal replacement therapy).

All statistical analyses were performed using statistical software SAS (version 9.3, SAS Institute Inc., Cary, NC, USA) or STATISTICA (version 9.1, StatSoft Inc., USA). Continuous variables were described as means (with standard deviation) or medians (with inter-quartile range), as appropriate, and categorical variables as percentages. The Wilcoxon rank sum test was used to compare urinary biomarker levels at each time point between patients with and without AKI. For statistical analysis of possible effect of the factor AKIN level and time or interaction of both factors with the NGAL concentration and NGAL amounts, a nonparametric two-factorial ANOVA with interaction term was performed . The significance level and the P values were adjusted using the Bonferroni-Holm method. Differences were considered statistically significant at a P value of less than 0.05.

## Results

Eighty one patients, 29 women (mean age 68 ± 9 years) and 52 men (mean age 67 ± 14) were included in the study. Coronary artery bypass grafting (GABG) was performed in 35 patients (16 female, 19 male), valve replacement in 19 patients (6 female, 13 male), and combined procedures in 27 patients (7 female, 20 male).

**Fig. 1:**
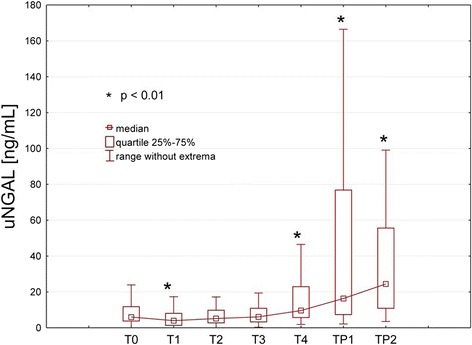
The median uNGAL concentrations of all study patients at the different measurement points. T_0_: baseline; T_1_: start CPB (*n* = 81); T_2_: 40 min CPB (*n* = 81); T_3_: 80 min CPB (*n* = 70); T_4_: 120 min CPB (*n* = 40); T_P1_: 15 min after CPB (*n* = 81); T_P2_: 4 h after admission to the intensive care unit (*n* = 81). *p < 0.01 if compared to T_0_

The concentration of uNGAL, decreased significantly at the beginning of CPB (T_1_) (*p* < 0.01 vs. T_0_) without further significant changes until 80 min on CPB (T_3_). At 120 min on CPB and thereafter uNGAL increased significantly (*p* < 0.01 vs. T_0_) (Fig. 1).

**Fig. 2:**
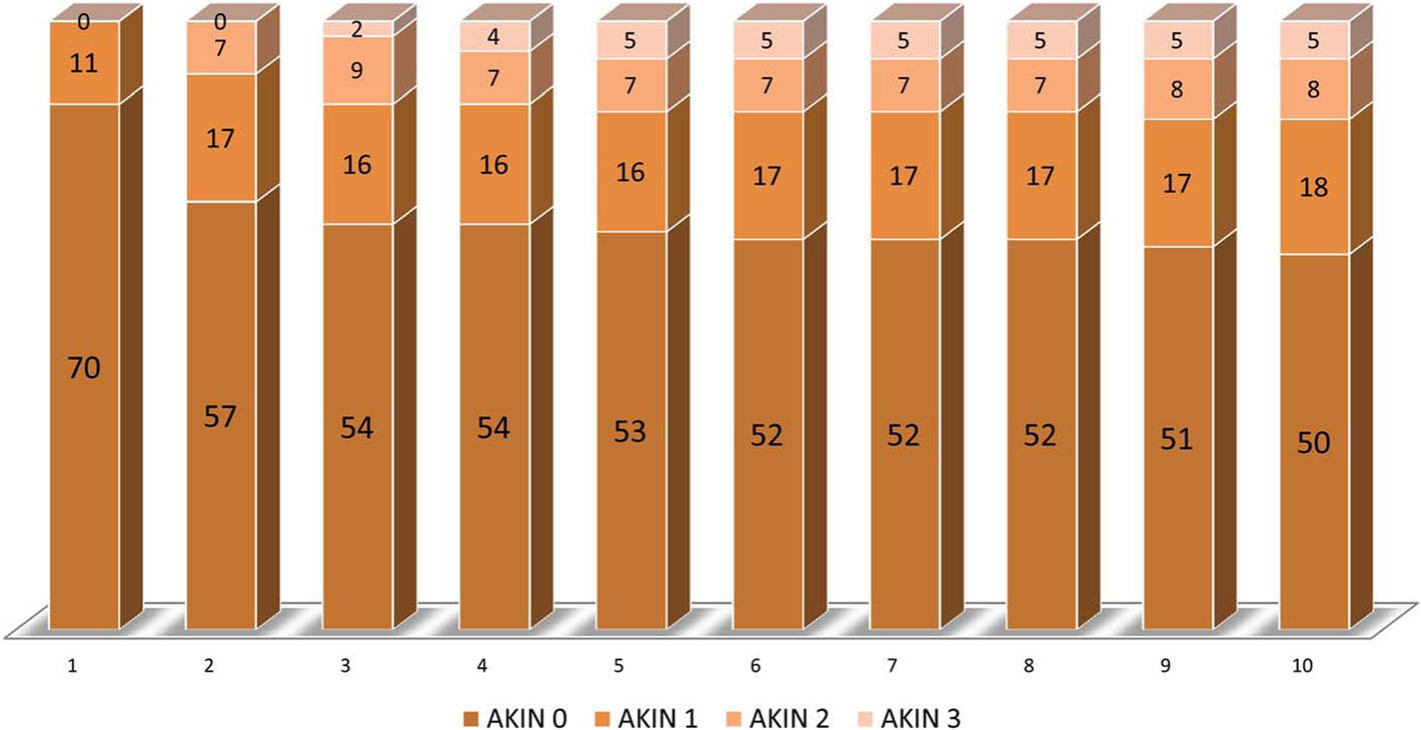
Number of patients in the corresponding AKIN groups over 10 days after surgery

Up to 10 days after surgery fifty patients (62%) had a normal renal outcome (AKIN level 0), 18 patients (22%) were in AKIN level 1, eight patients (10%) developed AKIN level 2, and five patients (6%) developed severe renal injury (AKIN level 3). Four patients of five in AKIN level 3 underwent hemodialysis. Most patients, 27 out of 31, developed the renal injury (AKIN level 1, 2, and 3) within the first four postoperative days. The clinical data of the patients did not show significant differences (Table 1). Nine additional patients (11%) developed renal failure after the 4^th^ postoperative day (Fig. 2). Three patients died postoperatively; one patient without and two patients with severe postoperative renal injury.

**Table 1:** Preoperative, operative, and postoperative clinical data of the patients after on-pump cardiac surgery according to AKIN levels on postoperative day 4

	AKIN level 0	AKIN level 1	AKIN level 2	AKIN level 3
Males/Females	30/15	15/6	5/3	2/5
Age (years)	62.9 ± 14.1	72.5 ± 9.3	70.5 ± 4.4	74.9 ± 3.3
Weight [16]	79.9 ± 16.2	80.9 ± 15.8	93.0 ± 25.6	83.8 ± 13.7
CPB duration (minutes)	139.3 ± 50.2	148.1 ± 50,8	152.9 ± 59.7	130.3 ± 30.9
Stay in OR (minutes)	249.7 ± 71.6	248.6 ± 73.4	267.3 ± 78.6	241.9 ± 28.9
Intraoperative urine output (ml/kg)	10.4 ± 15.8	7.2 ± 6.7	4.9 ± 3.8	18.3 ± 36.6
Early postoperative urine output (5 h) (ml/kg)	8.7 ± 4.7	7.4 ± 5.0	5.2 ± 4.8	5.7 ± 3.5
Peak free Hb in blood (mg/100 mL)	57.6 ± 29.0	59.3 ± 20.1	71.3 ± 23.2	62.5 ± 31.1
Peak free Hb in urine (mg/100 mL)	22.2 ± 38.3	13.8 ± 12.1	20.0 ± 16.9	34.0 ± 61.0
ICU stay (days)	2.5 ± 2.3	4.9 ± 6.3	4.3 ± 3.0	6.4 ± 3.8

*CPB* cardiopulmonary bypass, *OR* operating room, *Hb* hemoglobin, *ICU* intensive care unit

No significant differences in patient characteristics were noted between the groups with renal injury (AKIN levels 2 and 3) if compared to patients at AKIN level 0 and 1 (Table 2). The preoperative ARF-score was notably higher in patients who later developed kidney damage as in patients without severe renal injury (Table 3).

**Table 2:** Clinical data of the patients after CPB surgery classified to AKIN levels on postoperative day 4. Patients in AKIN level 0 + 1 did not receive specific renal treatment

	AKIN level 0 + 1	AKIN level 2 + 3	*p*-value
Patients	66	15	
Male gender (%)	45 (55.6)	7 (8.6)	0.117
Age (years)	66 (21–87)	73 (62–79)	0.071
Weight (kg)	80	89	0.087
CPB duration (minutes)	141.3	141.7	0.966
Intraoperative urine output (ml/kg)	9.1	11.1	0.672
Early postoperative urine output (5 h; (ml/kg)	8.0	5.5	0.094
ICU stay (days)	3.2	5.3	0.063
Hospital stay (days)	13.5	14.7	0.637

*CPB* cardiopulmonary bypass, *ICU* intensive care unit

**Table 3:** The preoperative values of the calculated Acute Renal Failure (ARF) scores, the creatinine, and the estimated glomerular filtration rate (GFR) in relation to the AKIN-levels classified on postoperative day 4. The calculated ARF score in AKIN-levels 2 + 3 were significantly higher than in AKIN-levels 0 + 1 (4.07 ± 2.46 vs. 2.91 ± 1.83, *p* = 0.043)

	AKIN 0	AKIN 1	AKIN 2	AKIN 3
ARF score	2.62 ± 1.74	3.52 ± 1.84	3.75 ± 1.85	4.43 ± 1.82
	2.91 ± 1.83		4.07 ± 2.46	
Creatinine (mg/dl)	0.99 ± 0.53	1.11 ± 0.33	1.03 ± 0.32	1.32 ± 0.64
	1.03 ± 0.48		1.17 ± 0.53	
GFR (ml/min/1.73 m^2^)	91.6 ± 41.9	69.8 ± 23.7	89.8 ± 34.7	43.3 ± 23.2
	83.3 ± 33.82		68.1 ± 26,83	

**Fig. 3:**
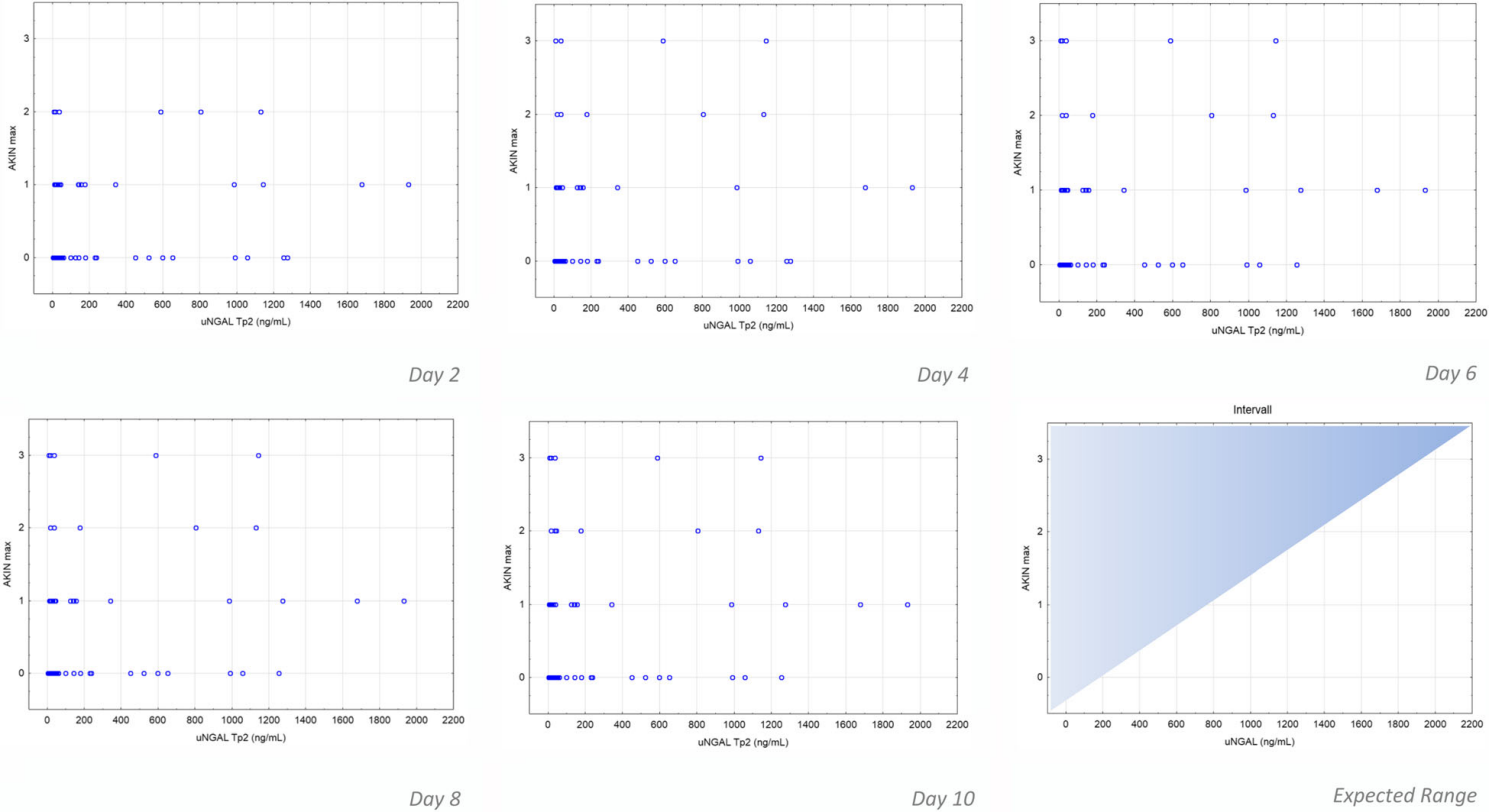
Correlation of uNGAL concentrations to corresponding AKIN levels on postoperative days 2, 4, 6, 8, and 10. The scatter-plots show uNGAL values at T_p1_ and T_p2_. A good prognostic valence is given if the values were mostly in the *left upper half* of the diagram (shadow area of index chart “expected range”)

**Fig. 4:**
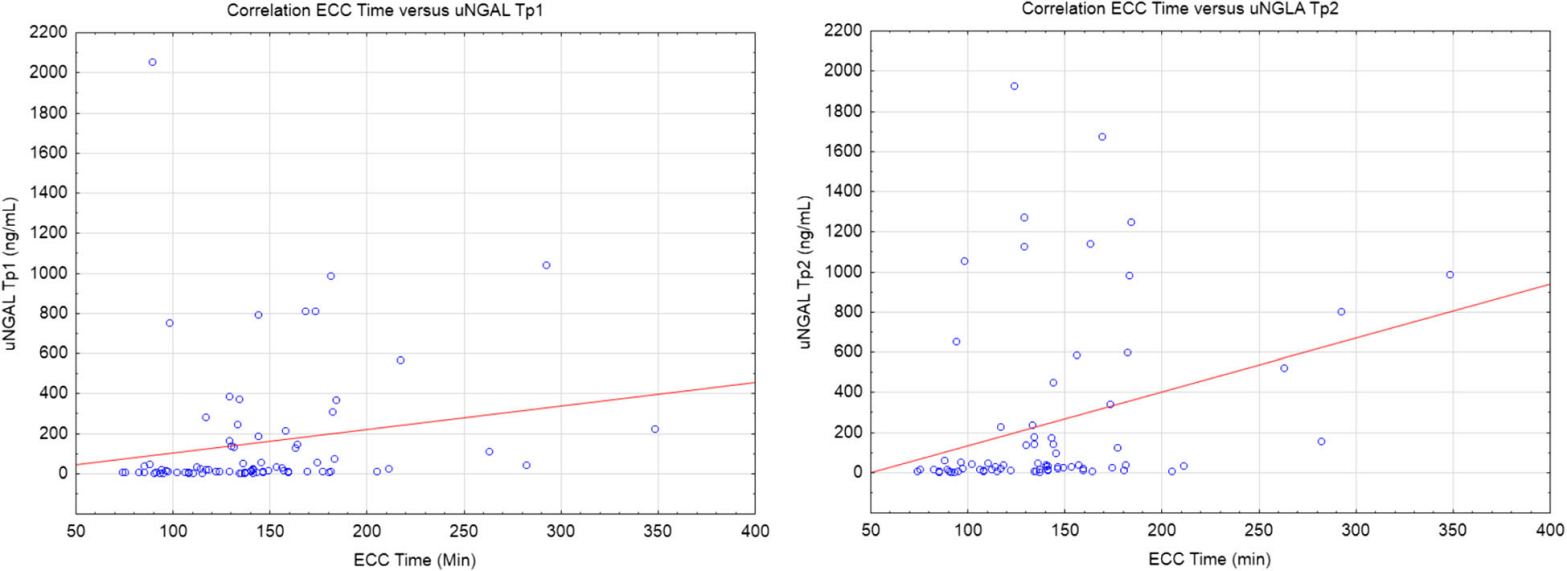
Correlation between CPB duration and uNGAL concentration at T_P1_ and T_P2_ (Pravis and Pearson; *r* = 0.177 and 0.308 respectively)

The relation of maximal uNGAL values (at T_p2_) and developed postoperative AKIN levels does not show significant correlation for prediction (Fig. 3). Also the relation of uNGAL values (at T_p1_ and T_p2_) to duration of CPB does not show significant correlation (Fig. 4).

Also in subgroup analysis (CABG, valve replacement, combined procedure, or other) there was no significant correlation between uNGAL value and AKIN level.

## Discussion

Acute kidney injury after cardiac surgery is a common complication and outcome may be devastating with a mortality-risk of up to 90% [6, 11, 16–19]. According to AKIN classification, changes in serum creatinine concentrations and reduced urine output define acute kidney injury. Therefore, changes of kidney function are detected with substantial delay. Changes of diuresis and/or serum creatinine levels that appear in the postoperative period can be directly ascribed to CPB, but also to various factors, as haemolysis, blood transfusions, volume deficit, haemodynamic instability, systemic inflammatory response (SIRS), or reduction of renal perfusion in the elderly [15, 20]. Nevertheless, early prediction of the occurrence of AKI after surgery is of crucial importance.

NGAL is a multi-potent 25-kDa protein mainly secreted by neutrophils, playing a fundamental role in iron metabolism [21, 22]. Although the dimeric NGAL is also expressed by other organs [23], the monomeric form is expressed by urothelial cells of the renal tubular system and has been specifically associated with epithelial stress [24, 25]. It is excreted in great amounts in urine [26] and in cases of ‘tubular stress’ it appears relatively early (60–120 min after stress induction) in the primary urine [27–29]. Although NGAL is an early predictive biomarker of acute kidney injury (AKI) some limitations of the certainty of NGAL’s predictive feature have been reported [6, 11, 12].

In general, the value of the costly analysis of several predictive biomarkers, such as uNGAL, interleukin-18 (IL-18), kidney injury molecule (KIM-1), N-acetyl-beta-D-glucosaminidase (NAG) and others, in comparison to the daily routine creatinine measurement is increasingly doubted. The aim of our study was to evaluate the use of uNGAL as a biomarker of AKI after cardiac surgery with CPB, assessing its concentration levels at seven crucial time points, i.e. before, during and after extracorporeal circulation. Additionally, we wanted to analyze the predictive power of uNGAL on severity of AKI. To the best of our knowledge, this is the first study which covers extensively the concentration fluctuations of uNGAL on pump in cardiac surgery.

If renal injury during extracorporeal circulation were caused by reduced renal perfusion, then an increase in NGAL-expression would be already expected in the intraoperative period. Our measurements showed a significant decrease of uNGAL at the beginning of cardiopulmonary bypass, whereas a significant increase of uNGAL was seen after 120 min of CPB. The initial uNGAL decrease is probably caused by haemodilution at the induction of anaesthesia. After 2 h on CPB and at the end of surgery and afterwards significant increase of uNGAL was seen. However, a statistical difference in uNGAL concentration was only observed between AKIN level 0 and AKIN level 1 (*p* < 0.05), so in patients without clinically relevant kidney injury. All other comparisons, i.e. between AKIN level 0 and AKIN level 2 or AKIN level 1 and AKIN level 2 did not demonstrate any statistical significant difference (*p* = 0.328 and *p* = 0.916, respectively).

Arteriosclerosis is a generalised disease which may affect many vessels and therefore CABG patients may have a higher risk for end organ failure based on the underlying disease. Although we would expect more kidney injury in CABG patients, we did not find significant differences between the procedure subgroups.

A limitation exists with sustained diuresis, as no determination of uNGAL concentrations can be made. The problem that arises is the possibility of miscalculations and misinterpretation of invalid results. Measuring uNGAL (ng/ml) in differential urine volumes by anuric and/or polyuric phases is less representative of the filtration and concentration efficacy of the kidneys as quantitatively measuring uNGAL per time interval (μg/min). Therefore, we have also compared the uNGAL concentrations to the uNGAL quantity per sample collection intervals and no differences were found.

Since NGAL participates in iron metabolism, free haemoglobin (Hb) in plasma and urine was measured but there was no correlation between the highest uNGAL concentrations, the peak free-Hb values, and the postoperative AKIN level (Table 1).

The onset of the renal injury during and after CPB is very difficult to be determined, even with the help of modern biomarkers. As several studies, also present study failed to demonstrate a correlation between peak uNGAL value, duration of CPB, and the occurrence of AKI [6, 11, 29–31]. The mean CPB duration in all our patients was 141 min; in the group of patients, who developed severe kidney injury (AKIN level 3), the mean CPB time was 130 min (Table 3). Moreover, a comparison of CPB duration between the groups of patients without postoperative essential renal injury (AKIN level 0, AKIN level 1) and with renal injury (AKIN level 2, AKIN level 3) revealed no significant difference (CPB time 141.7 min in AKIN 0/1 groups vs. 141.3 min in AKIN 2/3, *p* = 0.966).

In our study, increased uNGAL concentration was not a certain predictor of the degree of an upcoming renal injury. We expected to find good prediction of AKI within the first 4 days after CPB but there was for any time after CPB no correlation between uNGAL and postoperative AKIN classification level.

## Conclusion

The results of present study clearly demonstrate that the biomarker uNGAL failed to predict the onset and the severity of acute renal failure after cardiac operations with extracorporeal circulation. Further studies are needed to analyze possible markers predicting postoperative kidney injury.

## Abbreviations

AKI: Acute kidney injury
AKIN: Acute Kidney Injury Network criteria
CPB: Cardiopulmonary bypass
CVVH: Continuous venovenous hemofiltration
ICU: Intensive care unit
NGAL: Neutrophil gelatinase-associated lipocalin
sCr: Serum creatinine
SIRS: Systemic inflammatory response syndrome
uNGAL: Urinary neutrophil gelatinase-associated lipocalin
